# Geographic distribution and genetic characterization of poxviruses from human infections in Georgia, 2009–2014

**DOI:** 10.1007/s00705-020-04922-x

**Published:** 2021-03-21

**Authors:** Ekaterine Khmaladze, Matthew R. Mauldin, Davit Tsaguria, Mari Gavashelidze, Ketevan Sidamonidze, Tea Tevdoradze, Yu Li, Mary G. Reynolds, Paata Imnadze, Yoshinori Nakazawa

**Affiliations:** 1 Department of Virology, Molecular Biology and Genome Research, R. G. Lugar Center for Public Health Research, The National Center for Disease Control and Public Health (NCDC) of Georgia, Tbilisi, Georgia; 2 Poxvirus and Rabies Branch, Division of High-Consequence Pathogens and Pathology, US Centers for Disease Control and Prevention, Atlanta, USA

## Abstract

Anthrax is endemic in Georgia, as are multiple zoonotic poxviruses. Poxvirus-associated infections share some clinical manifestations and exposure risks with anthrax, and so it is important to distinguish between the two. With this in mind, an archived collection of anthrax-negative DNA samples was retrospectively screened for poxviruses, and of the 148 human samples tested, 64 were positive. Sequence analysis confirmed the presence of orf virus, bovine papular stomatitis virus, and pseudocowpox virus. This study provides evidence of previously unrecognized poxvirus infections in Georgia and highlights the benefit of the timely identification of such infections by improving laboratory capacity.

The family *Poxviridae* includes several species of zoonotic viruses, most of which are included in the genera *Orthopoxvirus* and *Parapoxvirus*. Among the currently recognized zoonotic orthopoxviruses (OPXV) are cowpox virus (CPXV), monkeypox virus (MPXV), and vaccinia virus (VACV). Zoonotic parapoxviruses (PPXV) include pseudocowpox virus (PCPV), bovine papular stomatitis virus (BPSV) and orf virus (ORFV). In humans, cutaneous lesions caused by many poxvirus infections are localized and self-limiting (except for MPXV and variola virus, which cause a generalized rash). The appearance of localized poxvirus-associated lesions can be similar to lesions of other cutaneous infections, such as cutaneous anthrax or leishmaniasis [1], which can confuse clinical diagnostic algorithms and, in many cases, cause poxvirus infections to go undetected.

Poxviruses were reported in Georgia as early as the 1980s [2, 3]. More recently, in 2013, a previously unrecognized member of the genus *Orthopoxvirus* (Akhmeta virus, AKMV) was determined to be the causative agent of cutaneous lesions on the hands of two cow herders [4]. During the investigation of these cases, samples from suspected anthrax cases that had previously tested negative for evidence of anthrax were examined for possible poxvirus etiology. This resulted in the retrospective identification of a third human case of AKMV from a sample collected in 2010. Concurrent serological investigations indicated the likely circulation of OPXVs in rodents and cattle in eastern Georgia [4].

Beginning in January of 2016, after the confirmation of poxviruses as etiological agents of human disease in Georgia, the National Center for Disease Control and Public Health of Georgia (NCDC) instituted a surveillance system for suspected human cases of poxvirus infections. The case definition for suspected poxvirus infections includes clinical manifestations that were consistent with poxvirus infections, but exposure data and positive laboratory test results are necessary for a case to be classified as probable and confirmed, respectively.

In the first twelve months of operation, 27 laboratory-confirmed human cases of PPXV infection were identified, 21 of which were originally misclassified as suspected anthrax [5]. Most PPXV cases (85%) reported contact with cattle, while the remaining 15% had contact with sheep. These results highlight the importance of understanding which poxviruses are circulating in Georgia and the potential risk factors for their transmission to humans. In this study, we used poxvirus detection assays to perform differential diagnosis on anthrax-negative samples and to investigate the genetic diversity and geographic distribution of poxviruses circulating in Georgia. We approached this by examining samples from cases in which anthrax had been ruled out, as well as cases reported via the poxvirus surveillance system, and sequencing and genetic analysis were carried out for the positive specimens.

We examined samples available through the archive of DNAsamples at NCDC collected via routine anthrax surveillance between 2009 and 2014. The original samples consisted of swabs collected from cutaneous lesions of patients clinically diagnosed with anthrax. Samples that had tested negative for *Bacillus anthracis* by culture and by PCRbased on both *B. anthracis* markers (BioFire Diagnostics, Target 2 and Target 3) for which the quantity/quality of sample was sufficient were selected for this study. In total, 148 available samples were examined for the presence of poxvirus DNA. The protocol underwent CDC human-subjects review and was determined not to be research involving human subjects, and as such, approval by an institutional review board and written informed consent were not required.

The B2L genes of 13 PPXV-positive samples collected from 2014 to 2016 from four regions of Georgia through the newly established poxvirus surveillance program were subsequently sequenced. The B2L gene was selected in order to (1) identify the species of PPXVs present in Georgia and (2) examine the potential distribution of different PPXVs. The selection of samples for sequencing was based on sample quality and the quantity of DNA available.

**Diagnostic testing:** DNA samples were extracted from the initially submitted human clinical specimens using a DNeasy Blood & Tissue Kit (QIAGEN, USA) according to the manufacturer’s instructions. A total of 148 DNA samples were examined by TaqMan-based quantitative real-time PCR (qPCR) for the presence of both OPXV DNA and PPXV DNA using an ABI 7500 Fast Real-Time PCR instrument (Applied Biosystems) and methods described previously [6, 7].

**Sequencing:** DNA sequence data were generated for the B2L gene (envelope protein) [8]. PCR master mix was prepared using 2X GC buffer I (25 μl per reaction), 2.5 mM dNTP mixture (8 μl per reaction), and Taq DNA polymerase 5 U/μl (0.5 μl per reaction) from the TaKaRa LA Taq with GC Buffer Kit (catalog no. RR02AG). Each reaction contained 2.5 μl of water, 10 mM primers (forward and reverse; 2 μl each), and 10 μl of template DNA. The total reaction volume was 50 μl. The thermocycler (GeneAmp PCR System 9700, Applied Biosystems) run conditions were as follows: an initial activation step of 1 min at 94°C; followed by 30 cycles of 94°C for 30 s, 60°C for 30 s, and 72°C for 2 min; and then a final extension step of 72°C for 5 min. A 593-bp PCR product was observed by standard gel electrophoresis. Primer dimers in the PCR product were removed by incubation with ExoSAP-IT at 37°C for 15 min, followed by incubation at 80°C for 15 min for enzyme inactivation. The purified amplicons were sequenced using a BigDye® Terminator v.3.1 Cycle Sequencing Kit (Applied Biosystems, Inc., Foster City,CA) with 2.5 μl of BigDye® Terminator v.3.1, 1 μl of 10 mM the respective forward and reverse primers (separately), 3.5 μl of water and 3 μl of template (15 ng/μl). The running conditions were as follows: an initial activation step of 1 min at 94°C, followed by 35 cycles of 96°C for 10 s, 55°C for 5 s, and 62°C for 1 min; and hold at 4°C. The final reaction products were purified using Performa® DTR Gel Filtration Cartridges (EdgeBio, San Jose, CA) according to the manufacturer’s instructions. The sequences were determined using a Model 3130xl Genetic Analyzer (Applied Biosystems, Foster City, CA).

**Genetic data analysis:** B2L gene sequences were proofed and aligned using Geneious v11.0.4 (Biomatters Ltd., Auckland, New Zealand). Reference sequences representing each recognized species in the genus *Parapoxvirus* whose members are known to infect terrestrial mammals (PCPV-GQ329669, GQ329670; ORFV- DQ184476, KF837136, AY386263, HM133903; BPSV-KM875470, KM875471; parapoxvirus of red deer [PVRD]- KM502564) and an appropriate outgroup sequence (UK squirrelpox virus HE601899) were selected. The most appropriate model of molecular evolution was determined using MEGA6 [9]. Bayesian inference analysis was performed independently for each locus using MrBayes v3.2.2 [10] with the following parameters: 20 million iterations, GTR+G model of molecular evolution, trees sampled every 2,000 iterations, and a 25% burn-in to generate a majority-rules consensus tree.

Results from the retrospective case investigation showed that 64 of 148 human samples tested were positive for either OPXV (n = 1) or PPXV (n = 63) (Table 1). PPXV-positive cases were detected in six of the eight regions from which specimens had been collected and in Tbilisi. The majority (87.5%) of positive cases were from Kvemo Kartli (56%) and Kakheti (17%), which also had the largest number of specimens submitted (Fig. 1). The preponderance of submissions is likely attributable to the foci of anthrax occurring in those regions at the time of specimen collection and submission [11]. One specimen, collected from Kutaisi, Vani Region, in 2010, tested positive for the presence of an OPXV. An investigation of the circumstances of this case has been published previously [4].

**Table 1:** Summary of human-derived specimens tested at NCDC for the presence of poxvirus DNA, 2010–2014 (number positive/number tested)

Region	2009	2010	2011	2012	2013	2014	Total	% positive by region
Imereti		0/1		2/2	1/1		3/4	75
Kakheti		0/3	1/4	2/6	7/20	1/2	11/35	31.4
Kvemo Kartli	1/1	2/8	6/10	14/22	12/21	1/1	36/63	57.1
Mtskheta-Mtianeti			0/2	1/3	1/3		2/8	25
Racha-Lechkhumi			0/1				0/1	0
Samegrelo-Zemo Svaneti				2/2	0/1		2/3	66.7
Samtskhe-Javakheti			0/1				0/1	0
Shida Kartli			1/3		0/3		1/6	16.7
Tbilisi	1/1	0/4	4/8	1/5	3/8	0/1	9/27	33.3
Total	2/2	2/16	12/29	22/40	24/57	2/4	64/148	43.2
% Positive by year	100	12.5	41.3	55.5	42.1	50	43.2

**Fig. 1:**
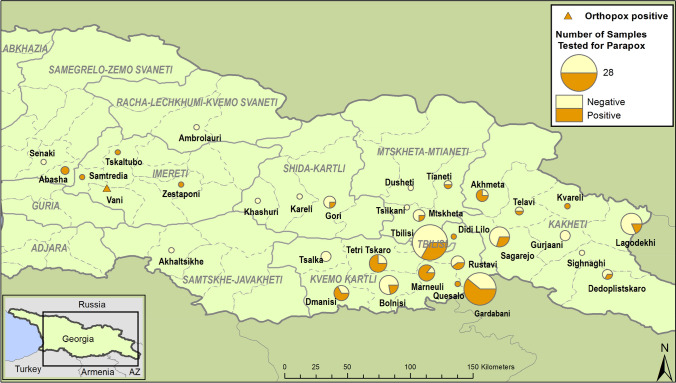
Geographic origin of specimens from cases in which anthrax was ruled out that were then tested for the presence of parapoxvirus DNA. Samples were collected from 2009 to 2014.

Among the specimens collected from 2014 to 16, following the formal implementation of surveillance for human poxvirus infection, thirteen were selected for single-gene (B2L) sequence analysis. All sequences generated in this study grouped within a monophyletic clade consisting of sequences for one of three PPXVs (Fig. 2). The majority were identified as belonging to species whose members are considered to be cattle-associated (11 PCPV, one BPSV) and one that more commonly affects small ruminants (ORFV; [12]). Sequence data were submitted to the GenBank database (accession numbers MH479407-MH479419).

**Fig. 2:**
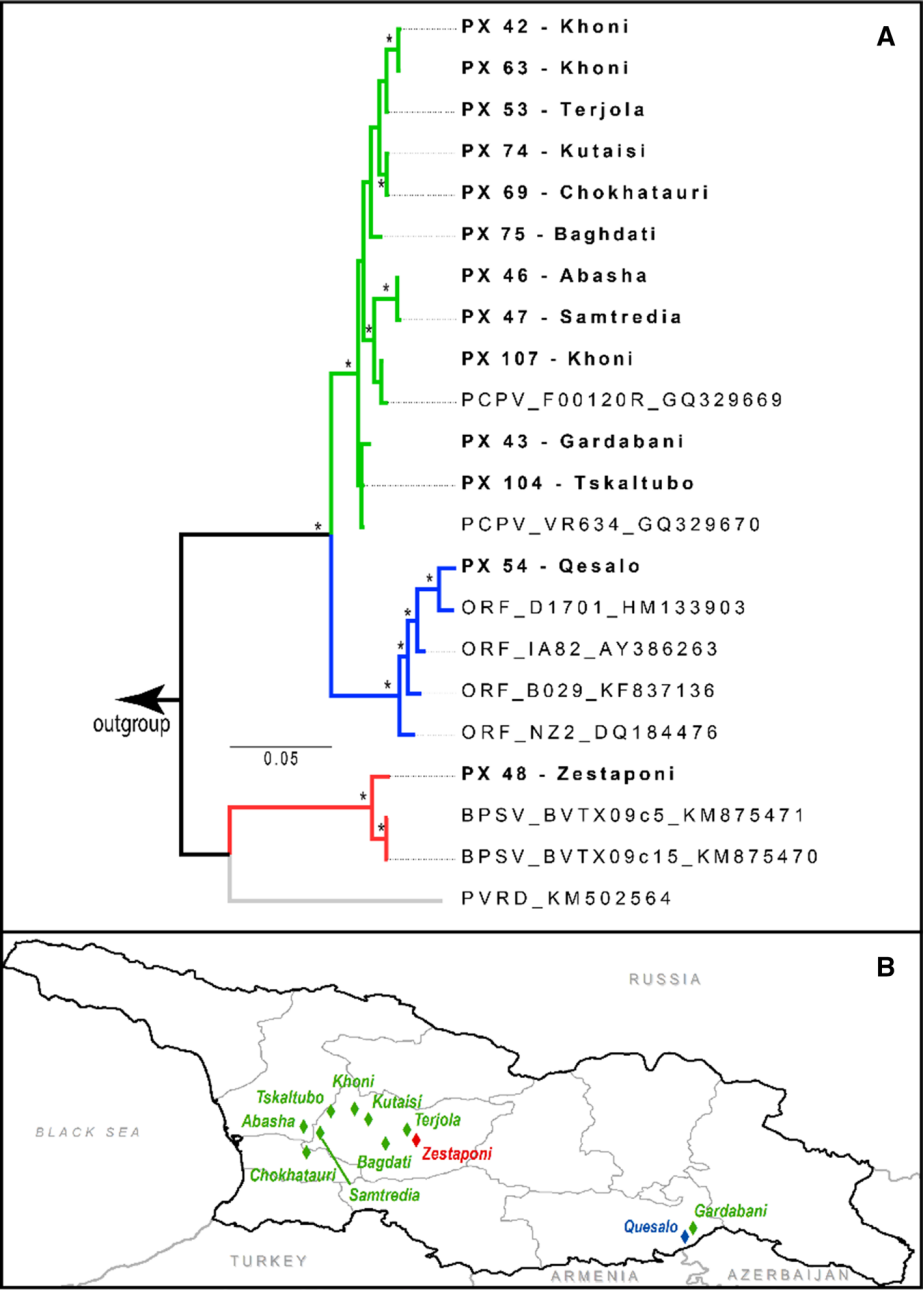
**a** Bayesian inference majority-rules consensus tree. *Indicates branches with 95% or higher Bayesian posterior probability (BPP) values. All study samples group with high statistical support with known parapoxviruses. Branch colors indicate the species of the parapoxviruses: green, *Pseudocowpox virus*; blue, *Orf virus*; red, *Bovine papular stomatitis virus*; gray, *Red deerpox virus*. Taxon labels for study samples are bolded and written as follows: sample ID—locality. Reference samples are written as follows: species identity_isolate name_GenBank accession number. *PCPV* pseudocowpox virus, *ORF* orf virus, *BPSV* bovine papular stomatitis virus, *PVRD* parapoxvirus of red deer. **b** A map of the country of Georgia with localities where the sequenced samples originated labeled and color-coded to match the appropriate species of PPV identified by phylogenetic analysis

Prior to the establishment of a surveillance system for human poxvirus-associated infections, cases could only be detected mainly via Georgia’s anthrax surveillance program (i.e., as cases excluded for anthrax). This circumstance probably biased the place and timing of the infections detected. For example, the largest proportion (66%) of retrospective samples examined were collected from 2012 to 2013, resulting in 53% PPXV-positive cases occurring during the study period. The large number of cases detected during this time period may be explained by heightened suspicion of anthrax, owing to a gap in the national anthrax vaccination program (from 2012-2013) followed by a larger-than-normal number of clinical samples from suspected anthrax cases submitted for laboratory testing. It is worth noting that regions where many PPXV cases were confirmed are also known to have endemic *B. anthracis* [13]*.* For example, Kvemo Kartli and Kakheti contain broad swaths of pastureland and have seasonal grazing for both sheep and cattle. PPXV detection via the dedicated poxvirus surveillance program continues in these areas, and cases continue to be seen in areas where vaccination of livestock for anthrax has been reinstituted [14]. Members of three species of zoonotic PPXVs were identified in specimens obtained from patients with cutaneous lesions in Georgia. Ongoing surveillance and PPXV species identification will aid in the creation of an unbiased assessment of the geographic distribution of PPXVs in Georgia and the risks that PPXVs pose to human health and agricultural productivity.

Since poxvirus infections are known to be directly linked to occupational exposure to infected animals, we suggest investigating occupational risk factors associated with their transmission. Furthermore, a surveillance system for poxviruses in Georgia through a 3-tiered disease surveillance program encompassing sentinel and passive surveillance plus active investigation of laboratory-confirmed infections should be considered. As a response to the outcome of our retrospective analysis of samples, we propose the extension of surveillance and prevention training to front-line medical personnel in public health centers and primary health care units throughout the country.

There were several limitations to our study. First, using a retrospective sampling method does not allow the results to be generalized to the entire Georgian population, and our findings do not represent the overall situation in the country. Furthermore, for sequence analysis, we were not able to use the same batch of DNA samples that were used for qPCR screening, since the quality and/or volume was not appropriate; therefore, the number of DNA samples for Sanger sequencing was low for evaluation of the geographic distribution of the viruses. Larger sections of the genome could provide greater resolution for examination of geographic patterns across the country and region.

In summary, we found that PPXVs are present in both eastern and western Georgia, where livestock (cattle, goats, and sheep) are raised. These results may explain the etiology of undiagnosed clinical cases seen in patients presenting with skin lesions during the sampling period. Poxvirus-associated infections have now been added to the notifiable diseases list in Georgia and to the Electronic Integrated Disease Surveillance System (EIDSS), and reporting of each suspected case has been required since January 2016 [5]. To avoid the development of antimicrobial resistance through misdiagnosis and inappropriate treatment with antibiotics, PPXV infections needs to be recognized early with correct treatment ensured. Predicting the emergence of disease is impossible without first knowing and understanding the range of endemic pathogens that are present.

## Data Availability

The genetic sequences generated and used for this report have been deposited in the GenBank database; accession numbers are provided in the text.
